# *FTO* genotype, dietary protein intake, and body weight in a multiethnic population of young adults: a cross-sectional study

**DOI:** 10.1186/s12263-018-0593-7

**Published:** 2018-02-20

**Authors:** David C. Merritt, Joseph Jamnik, Ahmed El-Sohemy

**Affiliations:** Department of Nutritional Sciences, University of Toronto, 150 College Street, Room 350, Toronto, Ontario M5S 3E2 Canada

**Keywords:** Nutrigenomics, *FTO*, Obesity, Protein, Diet, Weight loss, BMI

## Abstract

**Background:**

Variation in the fat mass and obesity-associated gene (*FTO*) has been associated with susceptibility to obesity, but the association appears to be modified by diet. We investigated whether dietary protein intake modifies the association between *FTO* variant rs1558902 and body mass index (BMI) and waist circumference in young adults (*n* = 1491) from the cross-sectional Toronto Nutrigenomics and Health Study.

**Results:**

Lifestyle, genetic, anthropometric, and biochemical data were collected and diet was assessed using a Toronto-modified Willett Food Frequency Questionnaire. General linear models stratified by ethnicity and adjusted for age, sex, and total energy intake were used to examine the association between FTO genotypes and measures of body weight, and whether protein intake modified any of the associations. East Asians who were homozygous for the rs1558902 risk allele (A) had a greater BMI (*p* = 0.004) and waist circumference (*p* = 0.03) than T allele carriers. This association was not observed in individuals of Caucasian or South Asian ancestry. Among East Asians, a significant FTO-protein interaction was observed for BMI (*p* = 0.01) and waist circumference (*p* = 0.007). Those with low protein intake (≤ 18% total energy intake) who were homozygous for the rs1558902 risk allele (A) had significantly higher BMI (*p* <  0.0001) and waist circumference (*p* = 0.0006) compared to carriers of the T allele. These associations were absent in the high protein intake group (> 18% total energy intake). Compared to Caucasians and South Asians, East Asians consumed a significantly higher ratio of animal-to-plant protein (*p* <  0.05).

**Conclusions:**

These findings suggest that high dietary protein intake may protect against the effects of risk variants in the *FTO* gene on BMI and waist circumference.

## Background

Despite increasing emphasis on the importance of health promotion, worldwide obesity rates and their associated health and economic burdens continue to rise. A recent systemic review of data collected from over 9 million individuals representing 199 countries reported that mean body mass index (BMI) has increased significantly worldwide since 1980 [1]. Associated with premature death, linked to cardiovascular disease, type 2 diabetes, and cancer, the widespread obesity epidemic has evolved into one of the most serious public health concerns of the century [2].

Obesity is known to have a significant genetic component alongside various lifestyle and environmental factors [3–6]. Socioeconomic status, education, physical activity, ethnicity, and dietary patterns have all been reported as significant environmental influences [5, 7, 8]. Strongly linked with the development of obesity, the fat mass and obesity-associated (*FTO*) gene was one of the first genetic loci identified as being associated with body weight [9–17]. Studies have reported a range of 0.25–0.41 kg/m^2^ increase in BMI per *FTO* risk allele, and a corresponding 20–40% increased risk of obesity [18]. Individuals homozygous for *FTO* risk variants are, on average, 3 kg heavier than those without such alleles [9]. Risk alleles are most prevalent in European populations (~ 42%) and least prevalent in African populations (~ 12%), accounting for 0.3 and 0.1% of total BMI variance, respectively [18, 19]. In Asian populations, the *FTO* risk allele frequency and explained variation in BMI is estimated to be 30 and 0.2%, respectively [20]. While there have been a large number of loci identified in addition to *FTO* which collectively explain a larger portion of total variation in BMI [21], the potential for effect modification by diet has been most extensively studied with *FTO*.

The association between *FTO* and dietary intake has been investigated in a number of studies. Variation in *FTO* has been associated with increased energy, fat, and protein intake [22–25]. Furthermore, it has been shown that the effects of several *FTO* polymorphisms on body weight can be modified by various dietary parameters [26–30]. Recently, a large randomized intervention trial investigated anthropometric measures and fat distribution in response to weight loss diets over a 2-year period [27]. Those who carried at least one copy of the *FTO* rs1558902 risk allele (A) experienced significantly greater reductions in body mass and fat distribution in response to a high-protein diet compared to T allele homozygotes, while this effect of *FTO* genotype was not observed in the low-protein treatment group. This interaction between dietary protein and *FTO* genotype on weight loss has been replicated in a population of obese adults in Spain [31]. However, the majority of participants in these studies were Caucasian, and it is unclear whether the *FTO* variants interact with dietary protein intake to influence measures of body weight in individuals of diverse ethnocultural backgrounds. The objective of the present study was to examine the association between genetic variation in *FTO* and measures of body weight and to determine whether protein intake modifies any observed associations in a cross-sectional population of ethnically diverse young adults.

## Methods

### Study population

Subjects (*n* = 1639) were individuals from the cross-sectional Toronto Nutrigenomics and Health (TNH) Study. Recruitment for the TNH study began in 2004 and ended in fall of 2010. The study was approved by the University of Toronto Ethics Review Board and informed consent was obtained from all individuals included in the study. Participants were males and females between 20 and 29 years of age. Individuals provided a self-reported account of their ancestry and were classified into one of four major ethnocultural groups—Caucasian, East Asian, South Asian, and “others”—as described previously [32]. All participants completed a 1-month 196-item semi-quantitative food frequency questionnaire (FFQ), a 63-item food preference checklist, and a general health and lifestyle questionnaire (GHLQ). The GHLQ included questions about physical activity, lifestyle habits, medication, dietary supplements, demographic status, dietary restrictions, education, and place of birth. Details of the Toronto-modified Willett FFQ used in the present study have been described previously [32]. Subjects provided a fasting blood sample from which DNA was isolated. Pregnant or nursing women and individuals who could not provide a blood sample were excluded from the study. We also excluded individuals with diabetes (*n* = 3), highly muscular subjects (*n* = 10), individuals missing *FTO* genotype, anthropometric, or dietary data (*n* = 20), and those belonging to the “other” ethnocultural group (*n* = 115). After exclusions, 1491 individuals (468 men and 1023 women) remained.

### Dietary assessment

A 1-month 196-item semi-quantitative FFQ (Toronto-modified Willet) was used to estimate each subject’s daily dietary protein intake. Subjects were given instructions and visual aids of common portion sizes to assist them in completing the FFQ. The base Willett FFQ has previously been validated for energy-adjusted protein intake against multiple 24-h recalls [33]. Protein intake was adjusted for total energy intake and expressed as percentage of total energy intake in all analyses in the present study.

### Anthropometric/biochemical measurements and genotyping

Anthropometric measurements including height, weight, blood pressure, and waist circumference were determined as previously described [32]. BMI (kg/m^2^) was calculated, and physical activity was measured by questionnaire and expressed as metabolic equivalent hours per week (MET), as described previously [32]. Blood samples were collected after a 12-h minimum fast by LifeLabs Medical Laboratory Services (Toronto, Canada) for DNA isolation and biochemical analysis using previously described methods [34]. Subjects were genotyped for rs1558902, a common single nucleotide polymorphism (SNP) in *FTO* at Princess Margret Hospital (Toronto, Canada) using Sequenom MassARRAY^®^ technology.

### Statistical analysis

All analyses were conducted using SAS Statistical Analysis Software v.9.2 (SAS Institute Inc., Cary, NC, USA). Variables that were not normally distributed were appropriately log_e−_ or square root transformed prior to analysis in order to satisfy the requirements of the statistical methods utilized, but the mean values and standard errors are reported in tables and figures without transformation to facilitate interpretation. Outcome variables BMI and waist circumference were log-transformed in all analyses. The *α* error was set at 0.05 and reported *p* values are two-sided.

Subject characteristics by rs1558902 genotype were compared using *χ*^2^ tests for categorical variables and ANCOVAs adjusted for age, sex, ethnicity, BMI, and total energy intake for continuous variables. The associations between rs1558902 genotype and BMI as well as waist circumference were explored using general linear models (GLMs) in each ethnocultural group. Analyses were adjusted for age, sex, and total energy intake. The Tukey-Kramer procedure was used to account for multiple comparisons when assessing differences in means between genotypes. Median energy-adjusted protein intakes were established for each ethnocultural subgroup and individuals were classified as either having “low” (≤ median) or “high” (> median) energy-adjusted protein intake. GLMs were then used to determine whether stratification by median values of energy-adjusted protein intake modified the association between rs1558902 genotype and BMI or waist circumference in each ethnocultural group. A test for the interaction between rs1558902 genotype and energy-adjusted dietary protein intake was performed on BMI and waist circumference. Macronutrient intakes were compared between those consuming “low” and “high” protein within each ethnocultural group. GLMs adjusted for age and sex were used to assess differences in energy-adjusted macronutrient intakes. GLMs adjusted for age and sex were also used to compare differences in the ratio of animal-to-plant protein consumption within each ethnocultural group.

## Results

Subject characteristics by FTO genotype are summarized in Table 1. The minor allele (A) frequency of rs1558902 was 30% for the total population, 42% in Caucasians, 13% in East Asians, and 29% in South Asians. Genotype frequencies were significantly different between ethnocultural groups, with the prevalence of risk allele (A) carriers being highest in Caucasians and lowest in East Asians (*p* < 0.0001). Significant associations were observed between rs1558902 and BMI (*p* = 0.02), physical activity (*p* = 0.005), homeostatic model assessment of insulin resistance (HOMA-IR) (*p* = 0.02), homeostatic model assessment of β cell function (HOMA-β) (*p* = 0.02), and triglycerides (*p* = 0.01) after adjusting for age, sex, ethnicity, BMI, and energy intake. No associations between rs1558902 and waist circumference (*p* = 0.07) or total energy intake (*p* = 0.17) were observed.

**Table 1:** Subject characteristics by *FTO* genotype

	rs1558902 genotype			
	TT	TA	AA	*p*
Subjects [*n*(%)]	762 (51)	569 (38)	160 (11)	
Age (year)	22.5 ± 0.1	22.8 ± 0.1	22.7 ± 0.2^a^	0.11
Sex [*n*(%)]				0.78
Male	233 (50)	184 (39)	51 (11)	
Female	529 (52)	385 (38)	109 (10)	
Ethnicity [*n*(%)]				< 0.0001
Caucasian	256 (34)	367 (48)	136 (18)	
East Asian	422 (76)	125 (22)	11 (2)	
South Asian	84 (48)	77 (44)	13 (8)	
BMI (kg/m^2^)	22.3 ± 0.1b	23.2 ± 0.2a	23.6 ± 0.3a	0.02
Systolic blood pressure (mmHg)	112.6 ± 0.4	114.7 ± 0.5	115.2 ± 0.8	0.77
Diastolic blood pressure (mmHg)	69.0 ± 0.3	69.7 ± 0.3	69.3 ± 0.6	0.73
Waist circumference (cm)	72.8 ± 0.3	75.1 ± 0.4	75.9 ± 0.7	0.07
Physical activity (METs)	7.7 ± 0.1b	7.6 ± 0.1b	8.4 ± 0.2a	0.005
Glucose (mmol/L)	4.79 ± 0.01	4.78 ± 0.02	4.77 ± 0.03	0.92
Insulin (pmol/L)	46.2 ± 1.0	47.6 ± 1.9	45 ± 2.6	0.39
HOMA-IR	1.39 ± 0.03	1.43 ± 0.06	1.34 ± 0.09	0.02
HOMA-β	107.2 ± 4.2	106.9 ± 3.8	101.7 ± 5.6	0.02
Cholesterol				
Total (mmol/L)	4.25 ± 0.03	4.26 ± 0.03	4.25 ± 0.06	0.93
HDL (mmol/L)	1.54 ± 0.01	1.54 ± 0.02	1.53 ± 0.03	0.28
LDL (mmol/L)	2.27 ± 0.02	2.28 ± 0.03	2.31 ± 0.05	0.71
Total/HDL (mmol/L)	2.89 ± 0.03	2.92 ± 0.04	2.88 ± 0.05	0.64
Triglycerides (mmol/L)	0.99 ± 0.02	0.97 ± 0.02	0.91 ± 0.03	0.01
hs-CRP (mg/L)	1.0 ± 0.1	1.5 ± 0.1	1.4 ± 0.2	0.59
Free fatty acids (μmol/L)	487.2 ± 9.1	483.8 ± 10.8	475.5 ± 20.8	0.82
Dietary intake				
Energy (kcal/day)	2033 ± 33	2116 ± 98	2017 ± 68	0.17
Protein (g/day)	89.9 ± 1.6	89.6 ± 1.7	84.0 ± 3.0	0.39
Total fat (g/day)	67.5 ± 1.2	71.0 ± 1.5	69.8 ± 3.0	0.96
Carbohydrates (g/day)	267.7 ± 4.5	279.6 ± 5.3	262.6 ± 8.7	0.52

Differences between groups assessed using *χ*^2^ test for categorical variables and ANCOVA adjusted for age, sex, ethnicity, BMI, and energy intake for continuous variables. Groups without a common letter differ after a Tukey-Kramer post hoc test (*p* < 0.05)

*METs* metabolic equivalent hours per week, *HOMA-IR* homeostasis model of insulin resistance, *HOMA-β* homeostasis model of β-cell function, *HDL* high-density lipoprotein, *LDL* low-density lipoprotein, *hs-CRP* high-sensitivity C-reactive protein

^a^Mean ± SE (all such values)

Subgroup analysis by ethnicity revealed a significant association between rs1558902 genotype and BMI (*p* = 0.004) and waist circumference (*p* = 0.03) in East Asians (Table 2), where risk allele homozygotes (AA, *n* = 11) had a higher BMI and waist circumference than T allele carriers (TT, *n* = 422; TA, *n* = 125). In Caucasians, TA heterozygotes (*n* = 367) at rs1558902 had a significantly greater waist circumference than TT homozygotes (*n* = 256), while AA homozygotes (*n* = 136) had a similar waist circumference to both TT (*n* = 256) and TA individuals. There was no association between rs1558902 genotype and BMI or waist circumference in South Asians. There was no significant interaction between *FTO* genotype and sex on BMI or waist circumference in any ethnocultural group. Further subgroup analyses were performed for Caucasians, East Asians, and South Asians to determine the relationship between protein intake, FTO genotype, and measures of obesity (Table 3). Median energy-adjusted protein intake in Caucasians, East Asians, and South Asians were determined to be 17, 18, and 17% of total energy intake, respectively. East Asians in the low-protein group (≤ 18% total energy intake) who were homozygous risk allele carriers (AA) had significantly higher BMI (*p* < 0.0001) and waist circumference (*p* = 0.0006) than carriers of the T allele. This association between rs1558902 genotype and BMI or waist circumference was not observed in the high protein intake group (*p* > 0.05). There were significant interactions between rs1558902 genotype and energy-adjusted protein intake on both BMI (*p* = 0.01) and waist circumference (*p* = 0.007) in East Asians. There was no significant interaction between rs1558902 genotype and dietary protein intake on BMI or waist circumference in Caucasian or South Asian individuals (*p* > 0.05).

**Table 2:** *FTO* genotype and measures of body weight stratified by ethnicity

	rs1558902 genotype			
	TT	TA	AA	*p*
Caucasians				
Subjects (*n*)	256	367	136	
BMI (kg/m^2^)	22.9 ± 0.2^a^	23.6 ± 0.2	23.4 ± 0.3	0.07
Waist circumference (cm)	74.7 ± 0.5a	76.4 ± 0.5b	75.6 ± 0.8ab	0.03
East Asians				
Subjects (*n*)	422	125	11	
BMI (kg/m^2^)	21.7 ± 0.1b	22.0 ± 0.2b	24.5 ± 1.2a	0.004
Waist circumference (cm)	71.0 ± 0.4b	71.4 ± 0.6b	77.0 ± 3.0a	0.03
South Asians				
Subjects (*n*)	84	77	13	
BMI (kg/m^2^)	23.7 ± 0.4	23.6 ± 0.5	24.8 ± 1.8	0.93
Waist circumference (cm)	76.4 ± 1.1	75.2 ± 1.5	77.2 ± 3.8	0.96

Differences between groups assessed using GLMs adjusted for age, sex, and energy intake

Groups without a common letter differ after a Tukey-Kramer post hoc test (p<0.05)

^a^Mean ± SE (all such values)

**Table 3:** *FTO* genotype and measures of body weight stratified by ethnicity and protein intake

	Low protein (≤ median intake)^a^				High protein (> median intake)^a^				
	rs1558902 genotype				rs1558902 genotype				Interaction
	TT	TA	AA	*p* ^b^	TT	TA	AA	*p* ^b^	*p* ^c^
Caucasian									
Subjects (*n*)	115	197	68		141	170	68		
Body mass index (kg/m^2^)	22.4 ± 0.3^d^	23.1 ± 0.2	23.1 ± 0.4	0.2	23.3 ± 0.3	24.1 ± 0.3	23.7 ± 0.4	0.17	0.95
Waist circumference (cm)	73.6 ± 0.7	76.2 ± 0.6	75.4 ± 1.1	0.07	75.5 ± 0.7	76.6 ± 0.8	75.9 ± 1.0	0.17	0.99
East Asian									
Subjects (*n*)	215	55	9		207	70	2		
Body mass index (kg/m^2^)	21.5 ± 0.2c	22.5 ± 0.3b	25.0 ± 1.3a	< 0.0001	21.8 ± 0.2	21.5 ± 0.3	22.4 ± 1.9	0.58	0.01
Waist circumference (cm)	70.6 ± 0.5b	72.0 ± 0.9b	78.9 ± 3.4a	0.0006	71.4 ± 0.5	71.0 ± 0.8	68.1 ± 1.3	0.34	0.007
South Asian									
Subjects (*n*)	42	40	5		42	37	8		
Body mass index (kg/m^2^)	23.8 ± 0.7	23.4 ± 0.7	24.4 ± 2.3	0.83	23.5 ± 0.5	23.9 ± 0.8	25.0 ± 2.7	0.86	0.86
Waist circumference (cm)	76.8 ± 1.7	74.4 ± 1.8	76.3 ± 5.2	0.75	76.0 ± 1.3	75.9 ± 2.5	77.8 ± 5.4	0.98	0.76

Groups without a common letter differ after a Tukey-Kramer post hoc test (p<0.05)

^a^Low and high protein intakes determined using ethnicity-specific medians: Caucasians (17% energy intake), East Asians (18% energy intake), South Asians (17% energy intake)

^b^Determined using GLMs adjusted for age and sex

^c^Interactions between rs1558902 genotype and energy-adjusted protein intake on markers of body mass determined using GLMs adjusted for age and sex

^d^Mean ± SE (all such values)

**Fig. 1:**
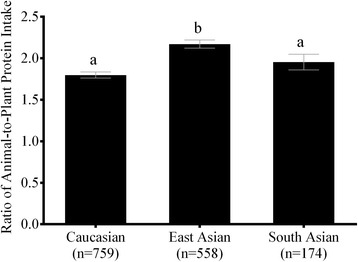
Ratio of animal-to-plant protein intake across ethnocultural groups. Differences in the ratio of animal-to-plant protein intake between groups were compared using GLMs adjusted for age and sex. Groups without a common superscript letter differ after a Tukey-Kramer post hoc test (*p* < 0.05)

Energy-adjusted macronutrient intakes in “low”- and “high”-protein consumers are shown in Table 4. High protein consumption was associated with significantly (*p* < 0.05) lower intake of total carbohydrates, sugars, and starches in all three major ethnocultural groups. High protein consumption was also associated (*p* < 0.05) with greater consumption of saturated fat in all ethnocultural groups. High protein intake was also associated (*p* < 0.05) with lower polyunsaturated fat intake in Caucasians, and greater total fat and monounsaturated fat in East Asians and South Asians. High total protein consumption was associated (*p* < 0.05) with greater intake of protein from animal sources across all ethnocultural groups, and lower intakes of protein from plant sources in Caucasians and East Asians. The ratio of animal-to-plant protein intakes across ethnocultural groups is shown in Fig. 1. East Asians had a higher ratio of animal-to-plant protein intake than Caucasians or South Asians (*p* < 0.05).

**Table 4:** Macronutrient intake in high- and low-protein consumers stratified by ethnicity

	Low protein (≤ median intake)^a^	High protein (> median intake)^a^	*p* ^b^
Caucasian (*n*)	380	379	
Carbohydrates			
Total carbohydrates (% energy intake)	54.3 ± 0.4^d^	50.0 ± 0.4	< .0001
Sugars^c^ (% energy intake)	25.0 ± 0.4	23.1 ± 0.3	0.0001
Fiber (g/day)	27.1 ± 0.7	26.2 ± 0.7	0.57
Starches (% energy intake)	24.2 ± 0.3	21.8 ± 0.3	< .0001
Carbohydrates from whole grains (% energy intake)	8.4 ± 0.3	9.2 ± 0.3	0.05
Fat			
Total fats (% energy intake)	30.5 ± 0.4	30.9 ± 0.3	0.70
Saturated fat (% energy intake)	9.4 ± 0.1	10.2 ± 0.1	< .0001
Monounsaturated fat (% energy intake)	12.5 ± 0.2	12.2 ± 0.2	0.38
Polyunsaturated fat (% energy intake)	5.9 ± 0.1	5.6 ± 0.1	0.01
Protein			
Total protein (% energy intake)	14.4 ± 0.1	19.1 ± 0.1	< .0001
Animal protein intake (% energy intake)	7.6 ± 0.1	12.7 ± 0.2	< .0001
Plant protein intake (% energy intake)	6.7 ± 0.1	6.4 ± 0.1	0.01
East Asian (*n*)	279	279	
Carbohydrates			
Total carbohydrates (% energy intake)	56.8 ± 0.4	49.6 ± 0.4	< .0001
Sugars^c^ (% energy intake)	24.7 ± 0.5	21.1 ± 0.4	< .0001
Fiber (g/day)	24.0 ± 0.9	20.8 ± 0.7	0.08
Starches (% energy intake)	27.5 ± 0.4	24.1 ± 0.4	< .0001
Carbohydrates from whole grains (% energy intake)	5.8 ± 0.3	6.1 ± 0.3	0.68
Fat			
Total fats (% energy intake)	28.4 ± 0.4	30.1 ± 0.3	0.0002
Saturated fat (% energy intake)	9.0 ± 0.1	9.8 ± 0.1	< .0001
Monounsaturated fat (% energy intake)	11.1 ± 0.2	11.6 ± 0.1	0.01
Polyunsaturated fat (% energy intake)	5.6 ± 0.1	5.6 ± 0.1	0.64
Protein			
Total protein (% energy intake)	15.5 ± 0.1	20.9 ± 0.1	< .0001
Animal protein intake (% energy intake)	9.1 ± 0.1	14.9 ± 0.2	< .0001
Plant protein intake (% energy intake)	6.4 ± 0.1	6.1 ± 0.1	0.01
South Asian (*n*)	87	87	
Carbohydrates			
Total carbohydrates (% energy intake)	60.5 ± 0.9	50.4 ± 0.9	< .0001
Sugars^c^ (% energy intake)	27.7 ± 0.8	21.3 ± 0.6	< .0001
Fiber (g/day)	23.2 ± 1.5	23.2 ± 1.5	0.77
Starches (% energy intake)	27.7 ± 1.0	24.4 ± 0.7	0.01
Carbohydrates from whole grains (% energy intake)	7.9 ± 1.1	8.2 ± 0.7	0.07
Fat			
Total fats (% energy intake)	27.2 ± 0.9	30.0 ± 0.7	0.01
Saturated fat (% energy intake)	8.6 ± 0.3	9.9 ± 0.3	0.004
Monounsaturated fat (% energy intake)	10.9 ± 0.6	11.6 ± 0.3	0.04
Polyunsaturated fat (% energy intake)	5.4 ± 0.2	5.7 ± 0.2	0.32
Protein			
Total protein (% energy intake)	14.0 ± 0.2	20.6 ± 0.3	< .0001
Animal protein intake (% energy intake)	7.4 ± 0.3	14.3 ± 0.4	< .0001
Plant protein intake (% energy intake)	6.6 ± 0.2	6.3 ± 0.2	0.39

^a^Low and high protein intakes determined using ethnicity-specific medians: Caucasians (17% energy intake), East Asians (18% energy intake), South Asians (17% energy intake)

^b^Determined using GLMs adjusted for age and sex

^c^Mean ± SE (all such values)

^d^Includes glucose, fructose, sucrose, lactose, and maltose

## Discussion

Although *FTO* is an established genetic susceptibility locus for obesity, the extent to which dietary factors modify this association has been unclear. Several studies have examined the effects of different dietary interventions on measures of body weight and composition, but the results remain inconsistent. We examined the interaction between *FTO* variant rs1558902 and dietary protein intake on BMI and waist circumference in a cross-sectional population of young adults of diverse ethnocultural backgrounds. Our findings indicate that protein intake modifies the effect of *FTO* risk variants in East Asian individuals, but not Caucasians or South Asians. East Asian individuals homozygous for the risk allele (A) of rs1558902 who had a low dietary protein intake had significantly higher BMI and waist circumference than T allele carriers. No association was observed between *FTO* genotype and BMI or waist circumference among those consuming higher amounts of protein. These findings suggest that dietary protein intake protects against the effect of the *FTO* risk variants on BMI and waist circumference. Our findings are based on East Asians living in North America, whereas almost all other studies evaluating *FTO* variants in Asian populations are based in Asia [35]. We did not observe a clear association between *FTO* and BMI or waist circumference, or any significant *FTO*-protein interaction on measures of body weight in Caucasians or South Asians.

The prevalence of the rs1558902 risk allele in the present study population is in agreement with previous reported values for Caucasian, East Asian, and South Asian populations [18–20]. The rs1558902 genotype has been robustly associated with body weight across multiple ethnicities and is in strong linkage disequilibrium with other *FTO* variants such as rs9939609 and rs9930506 [13, 18, 36, 37]. Using rs1558902 as a proxy for widespread variation in the *FTO* gene, we replicated the association between *FTO* risk variants and BMI [18]. Carriers of the risk allele had a significantly higher BMI (*p* = 0.02) than non-carriers and, although not significant, a similar trend was observed for waist circumference (*p* = 0.07).

Recently, there has been increased interest in determining whether dietary macronutrient composition interacts with variation in *FTO* to influence measures of body weight. The POUNDS LOST trial, a 2-year randomized weight loss intervention program, investigated the effects of different dietary treatments in a large population of obese individuals [27]. The authors observed that subjects with the risk allele (A) at rs1558902 who were placed on high-protein diets experienced greater positive changes in body composition and fat distribution than those on low-protein diets. No such effect of protein was observed among carriers of the T allele. These results suggest that dietary protein can mitigate the genetic risk associated with *FTO*. These findings were replicated in a recent weight loss trial which found that those with the *FTO* risk allele had greater weight loss and greater improvement in various metabolic parameters when placed on a high-protein diet [31]. However, a study of over 16,000 children from 14 study populations found that the adverse effects of variation in *FTO* on BMI were attenuated by low, rather than high, protein intake [38]. It is notable that the majority of subjects in the aforementioned studies were Caucasian, with only limited representation of other ethnocultural groups. Despite high prevalence of the risk allele and strong genetic effects reported in European populations, we were unable to detect an interaction between variation in *FTO* and protein intake on measures of body weight in young Caucasian individuals in the present study population. This is in agreement with analyses from the DiOGenes project, a European program focused on dietary components, genetics, and behavioral factors involved in the prevention of weight gain, which found no interaction between *FTO* and dietary protein intake on change in body weight or waist circumference at baseline or during the 6.8-year follow-up period [39]. They did, however, confirm an association between *FTO* variation and BMI and waist circumference at baseline. Furthermore, a recent meta-analysis evaluated 40 cross-sectional studies and found no interaction between *FTO* variants and protein intake on BMI [35]. That study did, however, identify an association between rs9939609 (or proxy SNPs) and higher dietary protein intakes, a relationship that was absent in the present investigation. However, the authors acknowledged that 87% of studies analyzed were based on Caucasian populations, and examinations of more diverse ethnic groups are clearly warranted.

We observed elevated BMI and waist circumference in East Asian risk allele (A) homozygotes at rs1558902 in the low protein intake group, but not in those consuming higher amounts of protein. The mean BMI of risk allele homozygotes in those consuming low amounts of protein was 25.0 ± 1.3 kg/m^2^, which makes them overweight, according to the United States Department of Health [40]. Furthermore, the average waist circumference for this group of participants was 78.9 ± 3.4 cm. Elevated BMI and waist circumference are established risk factors for cardiovascular disease [41, 42]. In the high protein intake group, all means were within normal ranges, and no significant differences in BMI or waist circumference were observed between genotypes. This would suggest that higher protein intakes may attenuate the association between *FTO* risk variants and adiposity and thus improve cardiovascular health outcomes in certain ethnocultural groups. We identified significant interactions between *FTO* genotypes and protein intake on both anthropometric outcomes in East Asians. Among East Asians, high total protein consumption was associated with lower intake of total carbohydrates, sugars, and starches. High total protein consumption was additionally associated with increased intake of total fats, saturated fat, and monounsaturated fat. High total protein consumption among East Asians was associated with greater animal protein intake, but less intake of protein from plant sources. Furthermore, compared to Caucasians and South Asians, East Asians consumed a significantly greater ratio of animal-to-plant protein. This suggests a potential effect of protein source on the observed interaction between *FTO* genotype and protein intake on body weight. Further investigation is needed to determine the clinical significance of these findings and potential applications as part of weight management interventions.

It has been established that *FTO* influences food intake rather than energy expenditure, yet many aspects of the association between variations in *FTO* and diet remain unclear. Many studies have identified an association between *FTO* risk variants and greater energy intake, especially in children and adolescents [23, 24, 38, 43, 44]. However, a large-scale multiethnic meta-analysis found an overall association between *FTO* risk variants and lower energy intake [35]. Interestingly, we observed no significant association between *FTO* variant rs1558902 and energy intake (*p* = 0.17). Additionally, contradictory to other studies that identified no association between variation in *FTO* and energy expenditure [22, 45], we identified a significant association between self-reported activity and rs1558902 genotype, where AA homozygotes were more physically active than T allele carriers in the overall population. However, this association was not observed in any ethnocultural group upon stratification by ethnicity (data not shown), and the self-reported measure of physical activity in the present study cannot be equated to direct measures of total energy expenditure.

Several limitations need to be considered when interpreting results from the present study. Although the total number of subjects included was relatively large, there were only 160 risk allele homozygotes (AA) for *FTO* variant rs1558902. Of these 160 individuals, 85% were Caucasian, so there was a limited subset of South and East Asian risk allele homozygotes. We were able to detect an association between rs1558902 and measures of body weight in East Asians in the low protein intake group; however, there were only two East Asian individuals with the AA genotype at rs1558902 genotype in the high protein intake group, and it is unclear if the lack of association in the high intake group was solely due to the modifying effects of protein intake, or whether a lack of statistical power could have played a role. Results of the present study should, therefore, be interpreted with caution. It is also possible that the lack of *FTO*-protein interaction on measures of body weight in Caucasians and South Asians was a result of FFQ-associated measurement error in assessing dietary protein intake, in addition to FFQ measurement error for individual food items as well as total energy intake. However, any measurement errors would likely occur equally across the different *FTO* genotypes and would not likely explain the observed associations. Moreover, despite its widespread use as a measure of body composition and adiposity, BMI does not take bone structure into account and cannot differentiate between lean and fat mass. For this reason, BMI is considered to be a poor surrogate measure of adiposity in some populations, which can lead to misclassification of obesity status [46]. While ethnicity-specific criteria for the classification of BMI have been suggested, BMI was treated as a continuous variable in the present study and all analyses were stratified by ethnocultural status, minimizing the potential effects of such differences. Waist circumference may be a better estimate of visceral body fat and can be a strong indicator of cardiovascular disease risk [47, 48]. In the present study, the analysis of BMI in conjunction with waist circumference minimized the risk of misclassification associated with the use of BMI as a measure of adiposity. Although analyses in the present study were adjusted for age, sex, and energy intake and stratified by ethnicity, it is possible that unaccounted for residual confounders influenced the results observed. Finally, the cross-sectional nature of our study precludes the establishment of causality in any of the associations we observed.

## Conclusions

Our findings suggest that dietary protein intake modifies the association between genetic variation in *FTO* and measures of body weight in certain ethnocultural groups. Higher dietary protein intakes might protect against the obesogenic effects of certain *FTO* genotypes and lead to improved individual metabolic profiles. The benefits of high-protein diets for weight management have been previously demonstrated [49, 50], and our results have further suggested a link between *FTO*, protein intake, and body weight. Elucidating the mechanism governing this gene-diet interaction is a clear direction for future research. Further studies should also focus on evaluating the viability of this nutritional strategy in personalized weight loss interventions.

## Abbreviations

BMI: Body mass index
FFQ: Food frequency questionnaire
*FTO*: Fat mass and obesity-associated gene
GHLQ: General health and lifestyle questionnaire
GLM: General linear model
HOMA: Homeostatic model assessment
MET: Metabolic equivalent hours per week
SNP: Single nucleotide polymorphism
TNH: Toronto Nutrigenomics and Health
